# Thermal Ablation Versus Surgical Resection for Intermediate-Size (3–5 cm) Colorectal Liver Metastases: Results from the Amsterdam Colorectal Liver Met Registry (AmCORE)

**DOI:** 10.3390/cancers18061017

**Published:** 2026-03-21

**Authors:** Madelon Dijkstra, Susan van der Lei, Hannah H. Schulz, Tineke E. Buffart, Rutger-Jan Swijnenburg, Martijn R. Meijerink

**Affiliations:** 1 Department of Radiology and Nuclear Medicine, Amsterdam University Medical Centers, 2333 ZG Amsterdam, The Netherlands; 2 Cancer Center Amsterdam, 1081 HV Amsterdam, The Netherlands; 3 Department of Medical Oncology, Amsterdam University Medical Centers, 2333 ZG Amsterdam, The Netherlands; 4 Department of Surgery, Amsterdam University Medical Centers, 2333 ZG Amsterdam, The Netherlands

**Keywords:** colorectal liver metastases (CRLM), thermal ablation, microwave ablation (MWA), radiofrequency ablation (RFA), surgical resection, intermediate-size metastases, local treatment

## Abstract

**Purpose**: Surgical resection has been the gold standard for colorectal liver metastases (CRLM) for decades. In recent years, thermal ablation has emerged as a first-line treatment option for small-size CRLM, while for intermediate-size lesions (3–5 cm), it is reserved for patients with unresectable disease. In this setting, thermal ablation has proven safe and effective, achieving durable local control (LC) in the majority of patients. This retrospective study compares oncological outcomes of thermal ablation versus surgical resection of intermediate-size (3–5 cm) CRLM. **Material and methods**: Patients treated with thermal ablation or surgical resection for intermediate-size CRLM between 2000 and 2025 were included. Baseline per-patient and per-procedure characteristics were compared across three groups: thermal ablation, surgical resection, and combined treatment. Per tumor characteristics were compared between thermal ablation and surgical resection. Primary outcomes included local tumor progression-free survival (LTPFS) and complication rates. Secondary outcomes were OS, distant progression-free survival (DPFS), LC, and length of hospital stay. Survival outcomes were analyzed using the Kaplan–Meier method; additionally, LTPFS was assessed using Cox proportional hazards regression models, with multivariable analyses performed to adjust for potential confounders. **Results**: A total of 320 patients with 448 metastases were included: 135 patients underwent thermal ablation, 156 underwent surgical resection, and 29 received combined treatment. LTPFS per tumor was significantly higher in the surgical resection group (HR 1.86, 95% CI 1.24–2.81, *p* = 0.0025), however, LC per tumor did not significantly differ amongst groups (HR 1.48, 95% CI 0.70–3.11, *p* = 0.307). Complication rates were significantly higher after resection (*p* < 0.001). OS and DPFS did not differ significantly between the three groups (*p* = 0.08 and *p* = 0.084). OS comparing only thermal ablation and resection was significantly lower in the thermal ablation group. Median hospital stay was 3, 5, and 7 days for the ablation, resection, and combined groups, respectively (*p* < 0.001). **Conclusions**: Thermal ablation offers a safe alternative to surgical resection for selected patients with intermediate-size (3–5 cm) CRLM, with higher treatment-site recurrence rates. With the option of repeat ablation, comparable local tumor control can be achieved. Improvements in local control with both modalities over time support the need for a prospective clinical trial.

## Simple Summary

This AmCORE-based retrospective study compared surgical resection with thermal ablation for the treatment of intermediate-size (3–5 cm) colorectal liver metastases. A total of 320 patients with 448 metastases treated between 2000 and 2025 were included. Local tumor progression-free survival and overall survival were significantly better following surgical resection, whereas ultimate local control—after accounting for retreatment—did not differ significantly between the two treatment modalities. Thermal ablation was associated with significantly fewer complications and a shorter hospital stay, supporting its safety profile for intermediate-size CRLM. Over time, substantial improvements in local tumor progression-free survival were observed for both treatments, with the most recent treatment period (2020–2025) showing smaller differences between modalities and no difference in ultimate local control. Thermal ablation offers a safe alternative to surgical resection for selected patients with intermediate-size (3–5 cm) CRLM, with higher treatment-site recurrence rates. With the option of repeat ablation, comparable local tumor control can be achieved. Improvements in local control with both modalities over time support the need for a prospective clinical trial.

## 1. Introduction

Colorectal cancer (CRC) is the third most common cancer worldwide, with an annual incidence of 1.9 million cases. In 2020, CRC accounted for 9.4% of all cancer-related deaths [1]. A major contributor to mortality in CRC patients is the development of colorectal liver metastases (CRLM), which affects approximately 50% of patients with CRC [2,3,4,5]. Without treatment, CRLM is a fatal condition, with 5-year overall survival (OS) rates of 0–3% [6,7,8]. Although systemic therapy alone can increase 5-year OS to 11%, it is generally only used in a palliative setting [6,7,8,9]. The only curative approach for achieving long-term disease control in CRLM is the local eradication of the metastases. Until recently, surgical resection was considered the gold standard, while thermal ablation and stereotactic body radiation therapy (SBRT) were typically reserved for patients with unresectable CRLM [3,4,10,11]. However, oncological outcomes following thermal ablation have significantly improved over the past decade, largely driven by procedural and technical advancements.

Technological advances, including higher-power generators, CTHA with or without contrast tagging of CRLM, and ablation confirmation software, have improved the ability to achieve adequate ablation margins and thereby enhanced oncological outcomes in small colorectal liver metastases. In addition, image-registration software enables more accurate evaluation of the ablation zone with intra-procedural margin assessment, allowing immediate re-ablation of areas at risk of recurrence, while other established developments—such as navigation systems and image-fusion techniques integrating MRI and/or ultrasound—further facilitate accurate antenna placement and assessment of technical success and ablation margins [12,13,14,15,16,17]. One key development is the use of CT hepatic arteriography (CTHA), which has significantly enhanced tumor visibility. This technique has not only enabled more precise antenna placement but has also facilitated the detection of additional, previously unknown lesions [13,18,19,20,21,22]. In parallel, the incorporation of confirmation software during CT-guided thermal ablation has been shown to improve the accuracy of achieving the intended minimal ablation margin, which is directly associated with lower rates of local tumor progression (LTP) [12,23,24]. Recent innovations, such as tumor tagging (t-CTHA), have further refined the assessment of minimal ablation margins by complementing confirmation software. In this technique, scheduled intra-arterial contrast administration prior to the ablation, with the goal of retaining contrast within the tumor vasculature at peak enhancement, effectively “tagging” the tumor. By persistent opacification of the tumor during and after ablation, t-CTHA may enable more accurate real-time evaluation of ablation margins [15,25]. Additionally, the development of higher-power systems capable of creating more spherical ablation zones has expanded treatment possibilities, allowing for larger ablation volumes without increasing complication rates [14]. The COLLISION trial, comparing surgical resection with thermal ablation for small-sized (0–3 cm) CRLM, demonstrated a high conditional likelihood that thermal ablation would be non-inferior to surgical resection in terms of OS. Following a preplanned interim analysis, the trial was halted early because thermal ablation was also associated with non-inferior local control and superior safety profiles [16]. As a result, thermal ablation is now included in multiple international guidelines as an equivalent alternative to surgical resection for patients with small-size CRLM [26,27,28].

In the current literature, larger tumor sizes (>3 cm) are strongly associated with exponentially reduced technical efficacy, resulting in higher rates of LTP following thermal ablation [29,30,31,32,33,34]. However, considering the substantial technical advancements of recent years, the question arises whether similar progress has been achieved for intermediate-size tumors and whether oncological outcomes such as LTPFS and OS have started to converge with those of surgery, despite parallel improvements in surgical techniques. Consequently, it may be reconsidered whether thermal ablation for intermediate-size tumors should still be regarded as a contraindication, as previously questioned [35,36]. The aim of this Amsterdam Colorectal Liver Met Registry (AmCORE) based study was to analyze the safety and efficacy of thermal ablation and/or surgical resection for intermediate-size (3–5 cm) CRLM.

## 2. Materials and Methods

This single-center retrospective AmCORE study was conducted at the Amsterdam University Medical Centers (Amsterdam, The Netherlands), a tertiary referral medical center for gastrointestinal and hepatobiliary malignancies. Data were extracted from the prospectively maintained AmCORE database, and reporting conforms to the ‘STrengthening the Reporting of OBservational studies in Epidemiology’ (STROBE) guidelines [37]. The Institutional Review Board approved the study protocol (METc 2021.0121).

### 2.1. Patient Selection and Data Collection

Per-patient, per-procedure, and per-tumor data for all patients treated with thermal ablation or surgical resection for intermediate-size (3–5 cm) CRLM were identified and extracted from the database. When necessary, additional information was obtained through retrospective review of the electronic medical records. Patients were classified into three groups: thermal ablation, surgical resection, or a combined group receiving both modalities, either during the same procedure or in separate procedures. For the per-patient analyses, patients presenting with multiple large tumors who underwent both surgical resection and ablation at any point during the course of the disease were classified in the combined group. For the per-procedure analyses, procedures in which a patient from the combined group received only ablation or only resection were assigned to the ablation-only or resection-only groups, respectively. Procedures in which both ablation and resection were performed during the same session were classified within the combined group.

### 2.2. Study Procedures

CRLM suitable for thermal ablation or surgical resection were identified on cross-sectional imaging and were evaluated in a multidisciplinary tumor board. The board was attended by, among others, (interventional) radiologists, hepatopancreaticobiliary and oncological surgeons, medical oncologists, and radiation oncologists. Imaging consisted of computed tomography (CT), magnetic resonance imaging (MRI), and [^18^F]-fluoro-2-deoxy-D-glucose (^18^F-FDG) positron emission tomography (PET)-CT scans and was assessed using the RECIST criteria [38]. Thermal ablation for intermediate-size (3–5 cm) CRLM was often performed in patients with unresectable disease. Resectability was assessed in accordance with current international and national guidelines, as well as institutional standards. Patients were classified as unresectable when surgical resection was not considered feasible or appropriate based on tumor distribution, insufficient future liver remnant, comorbidities, or other clinical considerations.

The use of (neo)adjuvant systemic therapy was not standardized in accordance with national guidelines [27]. Induction systemic therapy for downsizing and reducing procedural risk and systemic therapy for patients with potentially worse tumor biology (multiple intrahepatic recurrences < 6 months) were allowed.

### 2.3. Thermal Ablation

All ablations were in accordance with the instructions for use as provided by the manufacturer and the CIRSE quality improvement guidelines. As computational techniques for confirming tumor-free margins using image fusion and registration were not available during the earlier years of this study period, the documented minimum tumor-free margins for procedures performed more than fifteen years ago were visually estimated. Hereafter, CTHA was combined with rigid image fusion software that compares pre- and post-ablation CT scans to estimate ablation zone margins. In accordance with the CIRSE Standards of Practice for thermal ablation of liver tumors, the intended tumor-free ablation margin should ideally measure 0.5–1.0 cm at its smallest width; therefore, technical success in this study was defined as achieving a minimum margin of 5 mm [39]. A real-time CT-guided percutaneous approach was preferred in patients with no contraindications (e.g., <5 CRLM); all other patients received an open approach. The RF3000 generator with expandable LeVeen electrodes (RFA; Boston Scientific, Marlborough, MA, USA), the RITA system with compatible expandable electrodes (RFA; AngioDynamics BV, Amsterdam, The Netherlands), and Evident, Emprint, or EMPRINT^HP^ (MWA; Medtronic-Covidien, Minneapolis, MN, USA) or Solero (MWA; AngioDynamics BV, Amsterdam, The Netherlands) generators with compatible antennas were used for merely all thermal ablation procedures. Potential insufficient ablation margins were treated with overlapping ablations of residual tumor tissue.

### 2.4. Surgical Resection

Owing to the prolonged study period, surgical techniques for hepatic resection evolved substantially over time. Initially, resections were predominantly performed using an open surgical approach. With advancing surgical expertise and technological developments, minimally invasive techniques were progressively adopted, first through laparoscopic surgery and subsequently through robot-assisted surgery [40]. Robot-assisted procedures were performed using the *da Vinci* Surgical System (Intuitive Surgical, Sunnyvale, CA, USA). All procedures were performed under general anesthesia. Margin assessment was based on histopathology. The choice of surgical approach was based on prevailing standards of care at the time of treatment, as well as tumor characteristics and patient-specific factors. All surgical resections were performed under general anesthesia by board-certified surgeons with expertise in hepatobiliary surgery. Perioperative management followed institutional protocols consistent throughout the study period.

### 2.5. Follow-Up

A contrast-enhanced (ce) CT scan was performed <6 weeks after thermal ablation when incomplete thermal ablation was presumed. As recommended by local guidelines, ^18^F-FDG-PET CT scans were performed every 3–4 months in the first year, CT scans every 6 months in the second and third years, and every 12 months in the fourth and fifth years following both thermal ablation and surgical resection [27]. LTP was described as a solid and unequivocally enlarging mass or as focal ^18^F-FDG PET avidity at the surface of the ablated tumor. An additional MRI was performed in case of ambiguity regarding the presence of LTP or new tumors in the liver.

### 2.6. Statistical Analysis

Baseline characteristics concerning per-patient and per-tumor data were compared across the three groups: thermal ablation alone, resection alone, and both treatment modalities combined. Categorical characteristics were described as percentages and compared using the Pearson’s chi-square test, except for dichotomous characteristics, for which Fisher’s exact test was applied. Continuous variables were presented as mean (SD) or median (IQR) and compared using the independent t-test or Mann–Whitney U test. Additional Generalized Estimating Equations (GEE) were used to account for the correlation of multiple lesions within the same patient. Complications were graded according to Common Terminology Criteria for Adverse Events (CTCAE) 5.0 and compared using the chi-square test. Time-to-event outcomes (LTPFS, LC, OS, and DPFS) were defined from the date of treatment following SIO-DATECAN consensus guidelines and analyzed using the Kaplan–Meier method (log-rank test) [41]. Local control is equivalent to assisted technique efficacy, with the exception that repeat treatments using alternative modalities (e.g., other ablative techniques, radiation therapy, or surgical excision) are permitted. Deaths without distant local tumor progression (competing risk) were censored. The primary endpoint, LTPFS, was further evaluated using Cox proportional hazards regression, accounting for potential confounders in multivariable analysis. Cluster-robust standard errors were used for LTPFS and LC to account for the within-patient correlation of multiple lesions. Hazard ratios, robust standard errors, 95% confidence intervals, and *p*-values were calculated. Potential confounders were identified in univariable analysis (*p* < 0.100) and were consequently included in a multivariable analysis. Variables were considered as potential confounders if *p* < 0.050. When LTPFS differed 10% in the corrected model, the variables were considered actual confounders. Hazard ratios (HRs) and 95% confidence intervals (CIs) were calculated. All statistical analyses were performed using SPSS^®^ Version 24.0 (IBM^®^, Armonk, NY, USA) [42] and R version 4.0.3. (R Foundation, Vienna, Austria) [43].

## 3. Results

**Figure 1:**
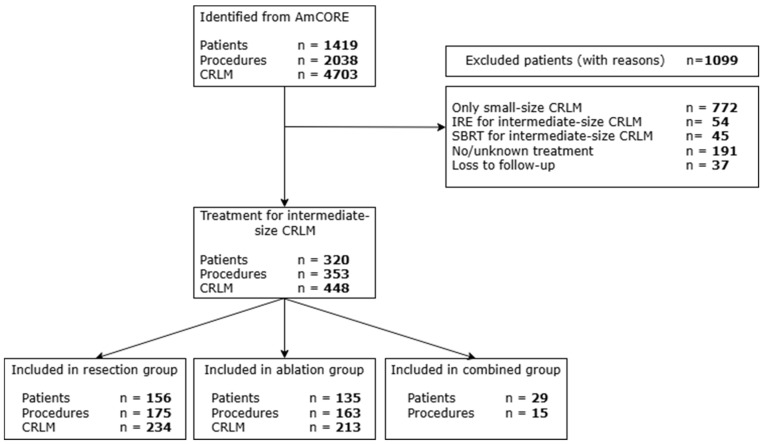
Flowchart of included and excluded patients.

A total of 1419 patients were identified in the AmCORE database. Of these, 1099 were excluded because they were treated solely for small-size tumors or received IRE or SBRT for intermediate-size tumors. A small number of additional patients were excluded due to missing or unknown treatment information or loss to follow-up (Figure 1).

### 3.1. Baseline Characteristics

A total of 320 patients who underwent local treatment for intermediate-size (3–5 cm) CRLM were identified from the prospective AmCORE database; 156 patients received surgical resection only, 135 received thermal ablation only, and 29 patients underwent both surgical resection and thermal ablation during the course of the disease. Within the combined group, 15 patients received surgical resection and thermal ablation during the same procedure; the remaining 14 patients underwent resection and ablation in separate procedures.

Patient- and disease-related characteristics are presented in Table 1. The cohort consisted of 63.1% males and 36.9% females, with a mean age of 65.7 years (SD 11.0). Comorbidities differed significantly between groups, with patients in the thermal ablation group more often presenting with minimal or major comorbidities. Primary tumor location and extrahepatic disease were well balanced across the treatment groups. Among patients with known mutational status, RAS mutations were present in 90.0% of the combined group, compared with 41.0% in the resection group and 25.5% in the ablation group (*p* = 0.001). The median follow-up for the entire cohort was 31.2 months.

A total of 353 local treatment procedures were performed: 175 surgical resections, 163 thermal ablations, and 15 combined procedures in which surgical resection and thermal ablation were performed during the same session. The number of tumors treated per procedure differed significantly, with combined resection and ablation performed in a single session treating a higher median number of tumors, 5 (IQR 3–6), compared to a median of 2 (IQR 1–4) in both the resection and ablation groups. The approaches and anesthesia techniques are described in Table 2. The majority of percutaneous procedures (79.6%) were CTHA guided.

A total of 448 tumors were included: 235 were treated with surgical resection and 213 with thermal ablation. Tumor-related characteristics are presented in Table 3. The median tumor size differed significantly, with 40 mm (IQR 34–52) for resected tumors and 35 mm (IQR 31–43) for ablated tumors (*p* < 0.001). Most resections were classified as minor (70.2%), with the remainder being major hepatectomies (29.8%). In the ablation group, a slight majority of tumors were treated with MWA (57.7%) rather than RFA (42.4%). Overall, the majority of tumors achieved margins > 5 mm (71.6%), with the proportion slightly higher in the ablation group (78.1%) than in the resection group (66.0%).

Additional analyses were performed to account for within-patient correlation. Using GEE to account for multiple lesions per patient, the associations remained statistically significant for both outcomes (*p* < 0.001 for tumor size and tumor margins), consistent with the results from the uncorrected analyses.

### 3.2. Overall Survival (OS)

The median OS for the entire cohort was 42.5 months (37.2–47.9). Median OS was 47.4 months (37.5–57.4) in the resection group, 38.5 months (32.0–45.0) in the ablation group, and 40.7 months (20.3–61.1) in the combined group.

**Figure 2:**
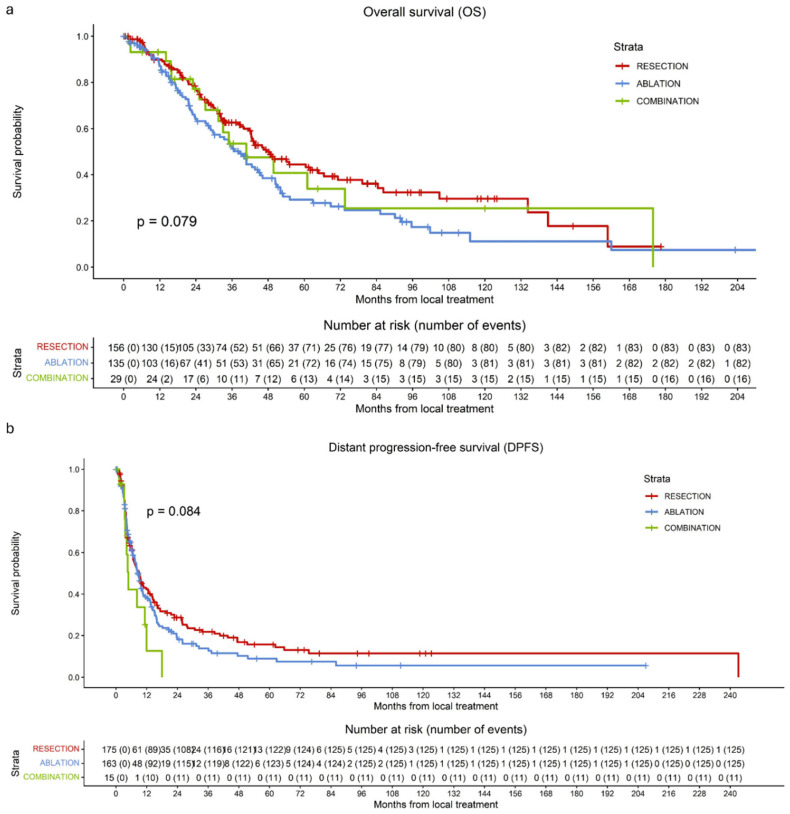
Kaplan–Meier curves of overall survival (OS) per patient (**a**) and distant progression-free survival (DPFS) per procedure (**b**) for patients undergoing surgical resection (red), thermal ablation (blue), or a combination of both modalities (green). (**a**) No significant difference in OS per patient was observed among the three treatment groups (*p* = 0.079). (**b**) No significant differences in DPFS per procedure were observed among the three treatment groups (*p* = 0.084).

During follow-up, 181 of 320 patients (56.6%) died: 83 of 156 (53.2%) in the resection group, 82 of 135 (60.7%) in the ablation group, and 16 of 29 (55.2%) in the combined group. No significant difference in OS was observed among the three treatment groups (*p* = 0.079) (Figure 2a). Across the entire cohort, 1-, 3-, and 5-year OS rates were 89.0%, 57.7%, and 37.9%, respectively. In the resection group, 1-, 3-, and 5-year OS rates were 89.8%, 62.6%, and 44.5%. Corresponding rates were 87.1%, 52.3%, and 29.2% in the ablation group and 93.3%, 53.5%, and 40.8% in the combined group. Sensitivity analyses did show a significantly higher OS when comparing only surgical resection and thermal ablation with an HR of 1.421 (95% CI 1.046–1.930; *p* = 0.025).

### 3.3. Distant Progression-Free Survival (DPFS) per Procedure

Median DPFS per procedure for the entire cohort was 8.7 months (7.4–10.0). Median DPFS was 9.0 months (6.5–11.4) in the resection group, 8.7 months (7.1–10.4) in the ablation group, and 4.8 months (3.7–5.9) in the combined group. Overall, 262 of 353 patients (74.2%) developed distant progression during follow-up: 126 of 175 (72.0%) in the resection group, 125 of 163 (76.7%) in the ablation group, and 11 of 15 (73.3%) in the combined group. There was no significant difference in DPFS among the three treatment groups (*p* = 0.084) (Figure 2b). DPFS rates are displayed in Table 4.

### 3.4. Complications and Length of Hospital Stay

Complication rates differed significantly among the treatment groups (Table 5; *p* < 0.001). Complications occurred in 77 of 175 (44.0%) resection procedures, 35 of 163 (21.5%) ablation procedures, and 10 of 15 (66.7%) combined procedures. The most frequent complications: in 6.5% of the procedures, patients developed abscesses requiring drainage; 2.8% experienced a biloma necessitating drainage, 2.8% developed pneumonia requiring antibiotics, and 2.8% had hemorrhage requiring an intervention.

Median length of hospital stay varied by treatment modality, with 5 days (IQR 5–9) in the resection group, 3 days (IQR 1–6) in the ablation group, and 7 days (IQR 5–19) in the combined group.

### 3.5. Local Tumor Progression-Free Survival (LTPFS)

**Figure 3:**
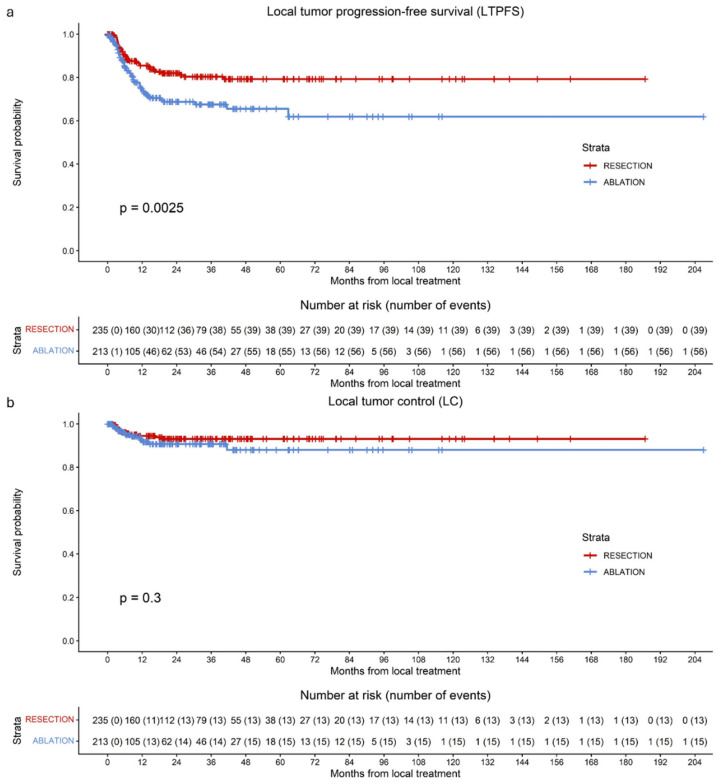
Kaplan–Meier curves of local tumor progression-free survival per tumor (LTPFS) (**a**) and local control per tumor (LC) (**b**) for patients undergoing surgical resection (red), thermal ablation (blue). (**a**) A significant difference in LTPFS per tumor between surgical resection and thermal ablation was observed (*p* = 0.0025). (**b**) No significant difference in LC per tumor between surgical resection and thermal ablation was observed (*p* = 0.3).

Overall, 95 of the 448 tumors (21.2%) developed LTP during follow-up: 39 of 235 (16.6%) in the resection group and 56 of 213 (26.3%) in the ablation group. A significant difference in LTPFS was observed between tumors treated with resection and those treated with ablation, favoring resection (HR 1.864; 95% CI 1.237–2.809; *p* = 0.003) (Figure 3a). Median LTPFS was not reached.

Altogether, 1-, 3-, and 5-year LTPFS rates were 80.3%, 74.6%, and 73.2%, respectively. In the resection group, 1-, 3-, and 5-year LTPFS rates were 85.5%, 80.4%, and 79.3%, respectively. Corresponding rates in the ablation group were 74.3%, 67.5%, and 65.6%, respectively.

All variables with *p* < 0.10 in univariable analysis remained significant in the multivariable model; consequently, no variables were removed by backward selection. After multivariable analysis, the HR was 2.644 (95% CI 1.566–4.465; *p* < 0.001) (Table 6).

As an additional analysis to account for the within-patient correlation of multiple lesions, cluster-robust Cox proportional hazards models were performed. These analyses confirmed that ablation was associated with a significantly higher risk of local tumor progression (HR = 1.864, 95% CI: 1.223–2.842, *p* = 0.004), consistent with the results from the uncorrected models.

### 3.6. Improvements over Time

**Figure 4:**
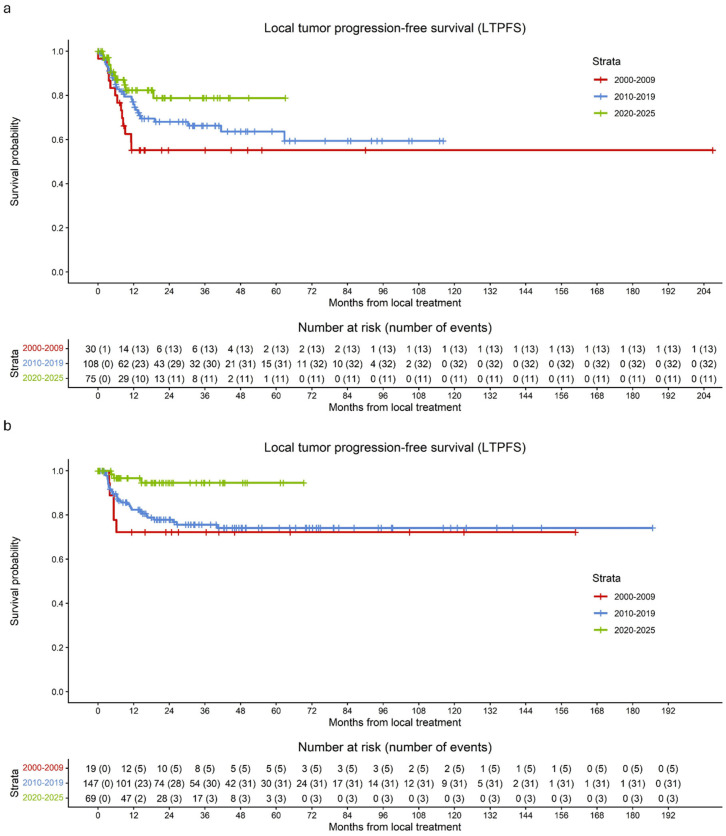
Kaplan–Meier curves of LTPFS per tumor following thermal ablation and surgical resection, stratified by time period: 2000–2009 (red), 2010–2019 (blue), and 2020–2025 (green). (**a**) The curves demonstrate an improvement in LTPFS per tumor after thermal ablation over time. (**b**) The curves demonstrate an improvement in LTPFS per tumor after surgical resection over time.

Overall, LTPFS after thermal ablation improved substantially over time (Figure 4a). Between 2000 and 2009, LTP occurred in 43.4% of ablated tumors, corresponding to a 1-year LTPFS of 55.2%. From 2010 to 2019, the LTP rate decreased to 29.6%, with a corresponding 1-year LTPFS of 75.9%. In the most recent period (2020–2025), LTP occurred in 14.7% of ablated tumors, with a 1-year LTPFS of 82.3%.

Additionally, LTPFS after surgical resection improved over time (Figure 4b). Between 2000 and 2009, LTP occurred in 26.3% of resected tumors, corresponding to a 1-year LTPFS of 72.2%. From 2010 to 2019, the LTP rate decreased to 21.1%, with a corresponding 1-year LTPFS of 82.4%. In the most recent period (2020–2025), LTP occurred in 4.3% of resected tumors, with a 1-year LTPFS of 96.7%.

### 3.7. Local Tumor Control (LC)

Overall, 28 of the 448 tumors (6.2%) demonstrated loss of local control during follow-up: 13 of 235 (5.5%) in the resection group and 15 of 213 (7.0%) in the ablation group. Median LC was not reached. No significant difference in LC was observed between tumors treated with resection and those treated with ablation, with an HR of 1.475 (95% CI 0.700–3.106; *p* = 0.307) (Figure 3b). The overall 1-, 3-, and 5-year LC rates were 93.3%, 92.1%, and 91.2%, respectively. In the resection group, 1-, 3-, and 5-year LC rates were 94.5%, 93.1%, and 93.1%, respectively. Corresponding rates in the ablation group were 91.6%, 88.1%, and 88.1%, respectively. As an additional analysis to account for the within-patient correlation of multiple lesions, cluster-robust Cox proportional hazards models were performed. These analyses confirmed no significant difference in LC (HR = 1.475, 95% CI: 0.6748–3.223, *p* = 0.300), consistent with the results from the uncorrected models.

**Figure 5:**
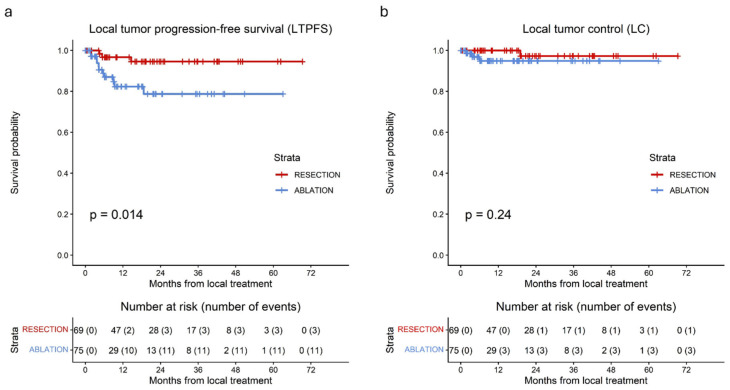
Kaplan–Meier curves for (**a**) LTPFS per tumor and (**b**) LC per tumor in patients treated between 2020 and 2025 with surgical resection (red) or thermal ablation (blue). (**a**) A significant difference in LTPFS was observed between resection and thermal ablation (*p* = 0.014). (**b**) No significant difference in LC was found between resection and thermal ablation (*p* = 0.24).

Additionally, Figure 5 illustrates LTPFS and LC for surgical resection versus thermal ablation during the period 2020–2025. Consistent with the overall LTPFS analyses, a significant difference was observed in favor of surgical resection (*p* = 0.014). In contrast, ultimate LC did not differ significantly between the two treatment modalities (*p* = 0.24).

## 4. Discussion

This AmCORE-based study compared surgical resection with thermal ablation for the treatment of intermediate-size (3–5 cm) CRLM. While LTPFS and OS were significantly better following surgical resection compared with thermal ablation, ultimate LC, after accounting for retreatment, did not differ significantly between the two treatment modalities. Thermal ablation was associated with a significantly lower complication rate, suggesting a safe profile for intermediate-size CRLM in addition to small-size CRLM. This study demonstrated substantial improvements in LTPFS following both resection and thermal ablation over time, with the most recent period (2020–2025) still showing persisting but reduced differences in LTPFS and no difference in eventual LC. While resection showed notable improvements over time, the findings of this study particularly emphasize the more pronounced advances in the effectiveness of thermal ablation as a treatment option for intermediate-size CRLM.

In the literature, tumor sizes above 3 cm are consistently associated with reduced technical efficacy and consequently higher LTP rates following thermal ablation [29,30,31,32,45,46,47]. One of the main challenges in larger tumors is achieving adequate ablation margins, a key prognostic factor of LTP and a predictor of technical success (A0 ablations), LTPFS, and LC. A minimum circumferential margin of 5 mm, and preferably >10 mm, is recommended to achieve these A0 ablation margins [34,39,48,49,50,51,52]. Image registration dedicated software provides better evaluation of the ablation zone, and intra-procedural software-based ablation margin assessment is crucial and offers a chance for immediate re-ablation of the areas at risk of recurrence [12,17]. Nevertheless, reported outcomes for intermediate-size CRLM vary substantially. A 2022 systematic review and meta-analysis, covering articles that describe procedures performed between 2000 and 2018, found per-procedure LTP rates varying between 11.1 and 62% with a median follow-up time of 25–36 months [53]. Eventual local control following repeat ablations was not reported specifically for intermediate-size CRLM in the included studies. Complications specific to intermediate-size CRLM were not separately described, though reported complication rates appeared comparable to those of the present cohort [53,54].

Correspondingly, Dijkstra et al. reported 1-, 3-, and 5-year LTPFS rates of 74.7%, 66.0%, and 66.0%, respectively, following thermal ablation of intermediate-size CRLM treated between 2000 and 2021. Notably, the study revealed significant improvements in both LTPFS and LC when comparing cohorts treated before and after 2010 (HR 0.315; 95% CI 0.127–0.781; *p* = 0.013) [33]. More recently, Wijnen et al. demonstrated that the hepatic artery catheter cone-beam (CB)CT-guided ablation (HepaCaGa technique) is safe and effective across a broad range of tumor sizes. The authors reported a 1-year LTPFS of 90% for 3–5 cm tumors, comparable to our 1-year LTPFS for thermal ablation performed between 2020 and 2025 [35]. Moreover, their LTPFS for intermediate-size tumors did not significantly differ from that of small-size tumors, suggesting that intermediate-size tumors should no longer be considered an absolute contraindication for thermal ablation [35,36].

Technological advances, such as ablation confirmation software, higher-power generators, and the use of CTHA with or without contrast tagging of the CRLM, have contributed to achieving adequate ablation margins and improved oncological outcomes for small-size CRLM [12,13,14,15,16]. These innovations may also explain the increasingly favorable outcomes being reported for intermediate-size tumors [33,35]. However, consensus regarding the optimal local ablative therapy, both thermal and non-thermal, for unresectable intermediate-size CRLM remains lacking, largely due to the absence of prospective randomized trials comparing thermal ablation with SBRT or IRE [11,53]. The ongoing phase II/III randomized COLLISION-XL trial (NCT04081168), which compares SBRT with MWA for unresectable intermediate-size CRLM, and the COLDFIRE-III trial (NCT06185556), which compares SBRT with IRE for CRLM up to 5 cm deemed ineligible for resection and thermal ablation, are expected to provide crucial evidence to inform clinical decision-making in this context [55].

The complication rates for both thermal ablation and surgical resection in our series are consistent with those reported in previously published studies. Although the COLLISION trial included small-size lesions, it reported a complication rate of 19% following thermal ablation and 46% following surgical resection, compared to 21.5% and 44.0%, respectively, in our series [16]. Both Qin et al. and Veltri et al. also did not find a significant correlation between complication development and lesion size. Qin et al. reported a mean lesion size of 1.8 cm in patients with complications versus 1.5 cm in those without (*p*  =  0.101) [56]. Similarly, Veltri et al. observed a mean lesion size of 2.7 cm in both groups [35,57]. More recently, Wijnen et al. reported even lower complication rates of 13% specifically for 3–5 cm CRLM following thermal ablation [35]. These findings raise an important question: now that sufficient ablation margins can be achieved in most cases and comparable local control is attainable with substantially fewer complications, should thermal ablation also be considered for resectable intermediate-size tumors?

Although RAS mutations were more frequent in the combined group, their prognostic significance in metastatic disease is context-dependent and largely restricted to left-sided tumors. Given the absence of stratification by tumor sidedness, the stronger association of poor OS with BRAF mutations (particularly in pMMR tumors), and the substantial proportion of missing molecular data, no definitive conclusions regarding tumor biology can be drawn. Furthermore, an advanced disease stage at diagnosis may reflect delayed detection rather than intrinsically aggressive disease. Aside from the higher LTP rates and reduced LTPFS observed in RAS-mutated tumors, there is no prospective evidence that this subgroup presents more frequently with multiple or larger lesions. [34,58,59].

The relatively large number of tumors included in this retrospective cohort supports the robustness of the analysis, although no formal power calculation was performed. However, the non-randomized study design represents a significant limitation, as it may have introduced selection bias and confounding. Although multivariable analyses were performed to adjust for potential confounders, residual confounding cannot be entirely excluded. Treatment strategies were discussed within multidisciplinary tumor boards based on local expertise, and patients in the thermal ablation group were classified as having unresectable tumors, which may further contribute to selection bias. Additionally, inclusion of patients treated more than 20 years ago introduces the possibility of population and historical biases, and the long follow-up period increases the likelihood of non-cancer-related deaths, which may have influenced OS. As this study included both patients who underwent open and robot resections, the median length of hospital stay was 5 days. However, it is noteworthy that, currently, 60% of resections are performed using robotic assistance, resulting in a median hospital stay of 3 days at the Amsterdam UMC.

A lower DPFS in the ablation group may indicate an underlying selection of biologically less favorable disease in these patients. This is not unexpected, as surgical resection was the preferred first-line treatment in this cohort, potentially resulting in baseline differences between groups that could partly explain the observed difference in overall survival. Alternatively, delayed or inferior local control in the ablation group, reflected by higher rates of local recurrence, may have contributed to earlier distant progression. Due to the retrospective nature of this study, causality cannot be established, and these hypotheses warrant confirmation in a prospective, ideally randomized, trial.

Notably, a higher proportion of ablation margins exceeding 5 mm was observed following thermal ablations compared to surgical resection; however, the ablation group exhibited higher LTP rates. This discrepancy may reflect an overestimation of ablation margins during earlier periods, likely due to the absence of modern confirmation and guidance technologies. Moreover, a direct comparison between adequate ablation margins assessed on post-ablation imaging and resection margins determined by pathological assessment is not feasible. The different assessment modalities and time points (imaging vs. histopathology; immediate vs. delayed) are not directly equivalent and may introduce bias in the comparison of margins between groups. Nevertheless, both sufficient ablation and resection margins demonstrate comparable predictive value with respect to the likelihood of LTP.

## 5. Conclusions

Thermal ablation offers a safe alternative to surgical resection for selected patients with intermediate-size (3–5 cm) CRLM, with higher treatment-site recurrence rates. With the option of repeat ablation, comparable local tumor control can be achieved. Improvements in local control with both modalities over time support the need for a prospective clinical trial.

## Figures and Tables

**Table 1:** Patient- and disease-related characteristics.

		Total N = 320	Resection N = 156	Ablation N = 135	Combination N = 29	*p*-Value
**Patient-related characteristics**						
Gender	Male	202 (63.1)	95 (60.9)	93 (68.9)	14 (48.3)	
	Female	118 (36.9)	61 (39.1)	42 (31.1)	15 (51.7)	0.082 ^a^
Age (years)	Mean (SD)	65.7 (11.0)	65.7 (10.9)	66.2 (11.4)	63.2 (9.8)	0.432 ^b^
ASA physical status	1	17 (5.4)	10 (6.4)	6 (4.5)	1 (3.4)	
	2	205 (64.7)	105 (67.3)	80 (60.6)	20 (69.0)	
	3	92 (29.0)	40 (25.6)	44 (33.3)	8 (27.6)	
	4	3 (0.9)	1 (0.6)	2 (1.5)	0 (0.0)	0.727 ^a^
	*Missing*	*3*	*-*	*3*	*-*	
Comorbidities *	None	141 (44.3)	74 (47.4)	49 (36.8)	18 (62.1)	
	Minimal	131 (41.2)	67 (42.9)	57 (42.9)	7 (24.1)	
	Major	46 (14.5)	15 (9.6)	27 (20.3)	4 (13.8)	**0.017 ^a^**
	*Missing*	*2*	*-*	*2*	*-*	
BMI	Mean (SD)	26.0 (4.1)	26.9 (5.1)	24.9 (4.0)	26.3 (4.6)	**0.048 ^b^**
**Disease-related characteristics at diagnosis**						
Primary tumor location	Right-sided colon	78 (24.5)	35 (22.4)	34 (25.4)	9 (31.0)	
	Left-sided colon	142 (44.5)	68 (43.6)	64 (47.8)	10 (34.5)	
	Rectum	99 (31.0)	53 (34.0)	36 (26.9)	10 (34.5)	0.538 ^a^
	*Missing*	*1*	*-*	*1*	*-*	
pT-stage	1	11 (3.9)	9 (6.3)	1 (0.9)	1 (3.8)	
	2	25 (8.8)	12 (8.4)	8 (7.0)	5 (19.2)	
	3	197 (69.6)	95 (66.4)	87 (76.3)	15 (57.7)	
	4	50 (17.7)	27 (18.9)	18 (15.8)	5 (19.2)	0.113 ^a^
	*Missing*	*37*	*13*	*21*	*3*	
pN-stage	0	107 (37.2)	54 (37.2)	42 (35.9)	11 (42.3)	
	1	109 (37.8)	52 (35.9)	46 (39.3)	11 (42.3)	
	2	72 (25.0)	39 (26.9)	29 (24.8)	4 (15.4)	0.854 ^a^
	*Missing*	*32*	*11*	*18*	*3*	
M-stage	0	160 (50.8)	85 (54.8)	57 (43.5)	18 (62.1)	
	1	155 (49.2)	70 (45.2)	74 (56.6)	11 (37.9)	0.072 ^a^
	*Missing*	*5*	*1*	*4*	*-*	
Molecular profile	RASwt/mut/unknown	72/47/201	36/25/95	35/12/88	1/9/19	**<0.001 ^a^**
	BRAFwt/mut/unknown	104/8/208	53/3/100	42/4/89	9/1/19	0.756 ^a^
	MSS/MSI/unknown	150/3/167	73/2/81	62/1/72	15/0/14	0.763 ^a^
Extrahepatic disease at diagnosis CRLM	No	293 (91.8)	141 (91.0)	123 (91.1)	29 (100.0)	
	Yes	26 (8.2)	14 (9.0)	12 (8.9)	0 (0.0)	0.243 ^a^
	*Missing*	*1*	*1*	*-*	*-*	

Categorical variables are reported as % of patients, continuous variables are reported as mean (SD), ^a^ = Pearson’s chi-square, ^b^ = One-way Anova, ASA = American Society of Anaesthesiologists score, CRLM = colorectal liver metastases * minor comorbidities included conditions that were well-controlled or unlikely to affect treatment (e.g., mild hypertension, controlled diabetes), whereas major comorbidities included conditions that could significantly influence treatment decisions or outcomes (e.g., severe cardiac disease, decompensated liver disease, advanced chronic kidney disease).

**Table 2:** Procedure-related characteristics.

		Total N = 353	Resection N = 175	Ablation N = 163	Combination N = 15	*p*-Value
**Procedure-related characteristics**						
Year	2000–2009	43 (12.2)	14 (8.0)	27 (16.6)	2 (13.3)	
	2010–2019	191 (54.1)	106 (60.6)	75 (46.0)	10 (66.7)	
	2020–2025	119 (33.7)	55 (31.4)	61 (37.4)	3 (20.0)	**0.031 ^a^**
Preprocedural chemotherapy	No	243 (68.8)	127 (72.6)	106 (65.0)	10 (66.7)	
	Yes	110 (31.2)	48 (27.4)	57 (35.0)	5 (33.3)	0.082 ^a^
Number of tumors	Median (IQR)	2 (1–4)	2 (1–4)	2 (1–4)	5 (3–6)	**0.011 ^b^**
Approach	Open	242 (68.6)	143 (81.7)	84 (51.5)	15 (100.0)	
	Laparoscopic	32 (9.1)	31 (18.3)	0 (0.0)	0 (0.0)	
	Percutaneous	79 (22.4)	0 (0.0)	79 (48.5)	0 (0.0)	
Anesthesia	General	308 (87.3)	175 (100.0)	118 (72.4)	15 (100.0)	
	Propofol	37 (10.5)	0 (0.0)	37 (22.7)	0 (0.0)	
	Dormicum	8 (2.3)	0 (0.0)	8 (4.9)	0 (0.0)	
Image-guidance technique	Conventional *	-	-	72 (20.4)	-	
	CTHA	-	-	281 (79.6)	-	-

Categorical variables are reported as % of patients, continuous variables are reported as median (IQR), ^a^ = Pearson’s chi-square, ^b^ = Kruskal–Wallis, * = intraoperative ultrasound or CT fluoroscopy, and CTHA = Computed Tomography Hepatic Arteriography.

**Table 3:** Procedure- and tumor-related characteristics.

		Total N = 448	Resection N = 235	Ablation N = 213	*p*-Value
**Tumor-related characteristics**					
Size (mm)	Median (IQR)	37 (32–47)	40 (34–52)	35 (31–43)	<0.001 ^a^
Technique/ablation modality	Minor resection	-	165 (70.2)	-	
	Major resection	-	70 (29.8)	-	
	RFA	-	-	90 (42.2)	
	RF3000™, LeVeen™	-	-	85 (39.9)	
	Cool-tip™	-	-	2 (0.9)	
	Starburst^®^ (RITA^®^)	-	-	2 (0.9)	
	Others	-	-	1 (0.5)	
	MWA	-	-	123 (57.7)	
	Evident™	-	-	2 (0.9)	
	Emprint™	-	-	65 (30.5)	
	Emprint™ HP	-	-	40 (18.8)	
	Solero™	-	-	6 (2.8)	
	Others	-	-	10 (4.7)	
Margin size (mm)	Margin-positive	31 (8.5)	25 (12.9)	6 (3.6)	
	1–5 mm	72 (19.8)	41 (21.1)	31 (18.3)	
	>5 mm	260 (71.6)	128 (66.0)	132 (78.1)	
	*Missing*	*85*	*41*	*44*	

Values are reported as % of patients, and continuous variables are reported as median (IQR), ^a^ = Mann–Whitney U test, RFA = radiofrequency ablation, and MWA = microwave ablation.

**Table 4:** Distant progression-free survival rates at 1, 3, and 5 years according to treatment group.

Treatment Group	1-Year DPFS (%)	3-Year DPFS (%)	5-Year DPFS (%)
Overall cohort	39.4	17.6	12.2
Resection	42.4	21.9	15.8
Ablation	38.0	13.8	9.0
Combined	12.7	0.0	0.0

Data are presented as percentages. DPFS indicates distant progression-free survival per procedure. Treatment groups include surgical resection, thermal ablation, and combined resection and ablation.

**Table 5:** Complications and length of hospital stay (CTCAE) [44].

	Total N = 353	Resection N = 175	Ablation N = 163	Combination N = 15	*p*-Value
Complications					
Grade 1	20 (5.7)	11 (6.3)	8 (4.9)	1 (6.7)	
Grade 2	31 (8.8)	21 (12.0)	7 (4.3)	3 (20.0)	
Grade 3	58 (16.4)	34 (19.4)	20 (12.3)	4 (26.7)	
Grade 4	14 (4.0)	11 (6.3)	2 (1.2)	1 (6.7)	
Grade 5	5 (1.4)	2 (1.1)	2 (1.2)	1 (6.7)	
Total	122 (34.6)	77 (44.0)	35 (21.5)	10 (66.7)	<0.001 ^a^
Length of hospital stay	5 (3–7)	5 (5–9)	3 (1–6)	7 (5–19)	<0.001 ^b^

Values are reported as % of procedures and median days (IQR), ^a^ = Pearson’s chi-square, ^b^ = Kruskal–Wallis.

**Table 6:** Univariable and multivariable Cox regression analysis to detect variables associated with local tumor progression-free survival (LTPFS).

		Univariable Analysis		Multivariable Analysis	
		HR (95% CI)	*p*-Value	HR (95% CI)	*p*-Value
Modality	Resection	Reference	**0.003**	Reference	**<0.001**
	Ablation	1.864 (1.237–2.809)		2.644 (1.566–4.465)	
**Patient-related characteristics**					
Gender	Male	Reference	0.775		
	Female	0.940 (0.616–1.435)			
Age		0.997 (0.978–1.016)	0.773		
ASA physical status	1	Reference	0.356		
	2	1.545 (0.563–4.238)			
	3	1.017 (0.349–2.964)			
	4	NA			
Comorbidities	None	Reference	0.583		
	Minimal	0.879 (0.568–1.359)			
	Major	0.717 (0.371–1.384)			
BMI		1.018 (0.975–1.064)	0.419		
**Disease-related characteristics**					
Primary tumor location	Right-sided colon	Reference	**0.049**	Reference	**0.023**
	Left-sided colon	0.542 (0.322–0.913)		0.420 (0.225–0.782)	
	Rectum	0.629 (0.390–1.014)		0.629 (0.356–1.112)	
pT-stage	1	Reference	0.452		
	2	4.004 (0.507–31.615)			
	3	2.708 (0.375–19.567)			
	4	2.313 (0.299–17.913)			
pN-stage	0	Reference	0.934		
	1	NA			
	2	NA			
M-stage	0	Reference	0.652		
	1	1.099 (0.730–1.654)			
Extrahepatic disease at diagnosis CRLM	No	Reference	0.608		
	Yes	0.790 (0.321–1945)			
**Procedure-related characteristics**					
Year	2000–2009	Reference	**0.002**	Reference	**0.029**
	2010–2019	0.644 (0.381–1.087)		0.784 (0.424–1.450)	
	2020–2025	0.284 (0.141–0.572)		0.373 (0.172–0.809)	
Preprocedural chemotherapy	No	Reference	0.618		
	Yes	0.895 (0.578–1.385)			
Number of tumors		0.954 (0.877–1.037)	0.270		
**Tumor-related characteristics**					
Tumor size		1.011 (0.99–1.024)	**0.077**	1.017 (1.000–1.034)	**0.044**
Margin size	Irradical	Reference	**<0.001**	Reference	**<0.001**
	1–5 mm	0.320 (0.156–0.655)		0.173 (0.080–0.373)	
	>5 mm	0.238 (0.132–0.429)		0.145 (0.077–0.272)	

HR = hazard ratio, 95% CI = 95% confidence interval, ASA = American Society of Anesthesiologists score, NA = insufficient group comparison.

## Data Availability

The data presented in this study are available upon request from the corresponding author.

## Abbreviations

The following abbreviations are used in this manuscript:

^18^F-FDG	[^18^F]-fluoro-2-deoxy-D-glucose
AmCORE	Amsterdam Colorectal Liver Met Registry
COLLISION	Colorectal liver metastases: surgery versus thermal ablation
COLLISION XL	Unresectable colorectal liver metastases: stereotactic body radiotherapy versus microwave ablation
Ce	Contrast-enhancement
CI	Confidence interval
CRC	Colorectal cancer
CRLM	Colorectal liver metastases
CT	Computed tomography
CTCAE	Common Terminology Criteria for Adverse Events
DPFS	Distant progression-free survival
HR	Hazard ratio
IRE	Irreversible Electroporation
LC	Local Tumor Control
LTP	Local tumor progression
LTPFS	Local tumor progression-free survival
MRI	Magnetic resonance imaging
MWA	Microwave ablation
OS	Overall survival
PET	Positron emission tomography
RECIST	Response Evaluation Criteria in Solid Tumors
RCT	Randomized Controlled Trial
RFA	Radiofrequency ablation
RR	Risk Ratio
SABR	Stereotactic Ablative Body Radiotherapy
SD	Standard Deviation
STROBE	Strengthening the Reporting of Observational Studies in Epidemiology
